# Relationship Between Neck Circumference and Risk Factors of Metabolic Syndrome in a Bushehr Elderly Health Study

**DOI:** 10.7759/cureus.40419

**Published:** 2023-06-14

**Authors:** Mohammadreza Kalantarhormozi, Marziyeh Bagheri, Maryam Marzban, Tara Motamedi, Azam Amini, Mehdi Mahmudpour, Mohamad Gholizadeh, Akram Farhadi, Iraj Nabipour, Afshin Ostovar, Bagher Larijani, Tahere Khayyati

**Affiliations:** 1 The Persian Gulf Tropical Medicine Research Center, The Persian Gulf Biomedical Sciences Research Institute, Bushehr University of Medical Sciences, Bushehr, IRN; 2 Internal Medicince, Los Angeles Biomedical Research Institute, Los Angeles, USA; 3 Osteoporosis Research Center, Endocrinology and Metabolism Clinical Science Institute, Tehran University of Medical Sciences, Tehran, IRN; 4 Endocrinology and Metabolism Research Center, Endocrinology and Metabolism Clinical Science Institute, Tehran University of Medical Sciences, Tehran, IRN

**Keywords:** bushehr geriatric health cohort, elderly, neck circumference index, metabolic syndrome, anthropometry

## Abstract

Metabolic syndrome includes a set of metabolic disorders such as obesity, high blood pressure, hypertriglyceridemia, lipid disorders, and glucose intolerance. In this cross-sectional (descriptive-analytical) study, 2,426 people were selected from the 60 years old and above population of Bushehr for a second-phase investigation of the relationship between neck circumference (NC) and cardiometabolic risk factors in the elderly people. The data (mean and standard deviation) were analyzed using STATA MP Version 15 software. The results of the study showed that the average age of all elderly participants in the study was 69.34 ± 6.39 years. The mean and standard deviation of the NC index in men, women, and all participants were 39.31 ± 2.89, 34.86 ± 2.84, and 37.00 ± 3.62, respectively. The mean and standard deviation of most laboratory indicators (triglyceride [TG], total cholesterol [TC], low-density lipoprotein [LDL], high-density lipoprotein [HDL]) were significantly higher in women, and there was no significant difference in fasting blood glucose (FBG) between men and women. NC index in the total population was significantly associated with all risk factors of metabolic syndrome (body mass index, systolic blood pressure, diastolic blood pressure) and laboratory indicators (FBG, TG, TC, LDLC, and HDL). The present study shows that the NC index can be a good predictor for the diagnosis of metabolic syndrome and visceral adipose tissue in the elderly.

## Introduction

The increase in human life expectancy has not progressed proportionally to the increase in healthy living habits. With the increase in the proportion of the elderly population, there is a natural increase in the prevalence of age-related disorders, such as Alzheimer's disease, type 2 diabetes, metabolic syndrome, weakness, and various other disorders [1]. Metabolic syndrome, also known as insulin resistance syndrome and syndrome X, is a group of interrelated disorders including insulin resistance, glucose intolerance, obesity, hyperlipidemia, and hypertension. The risk factors for metabolic syndrome include overweight, obesity, inactivity, and genetic predisposition. The prevalence of this disease has been reported differently in various countries. The prevalence is around 25% in the Middle East and between 6% and 42% in Iran. Various studies have reported the relationship between visceral fat and the incidence of metabolic syndrome [2].

Cardiometabolic indices

Cardiometabolic risk factors (CMRs) are a combination of conventional cardiovascular risk factors, such as high blood pressure, high cholesterol level, smoking, and metabolic risk factors, such as obesity, high blood sugar, and insulin resistance. Therefore, CMRs include obesity (abdominal or central), high triglycerides, low high-density lipoprotein (HDL), high blood pressure, and high blood sugar [3]. Obesity affects people of all ages, genders, ethnicities, and socioeconomic statuses [4]. Obesity is considered an important risk factor for cardiovascular diseases [5]. Central obesity is the main feature of the syndrome particularly, although patients with normal weight may also have insulin resistance and metabolic syndrome. Waist circumference (WC) is used to measure central obesity [4]. On the other hand, the fat in the upper part of the body correlates strongly with glucose intolerance, hyperinsulinemia, diabetes, hypertriglyceridemia, and increased risk of cardiovascular diseases, of which the neck skin fold and neck circumference (NC) are the indices [6]. Fat around the neck is a unique area for evaluating fat tissue in the upper body [5]. NC measurement is a simple screening for obesity and overweight in patients. Individuals with an NC of less than 37 in men and less than 34 in women have a lower chance of developing metabolic syndrome [7].

There are different methods such as computed tomography (CT), magnetic resonance imaging (MRI), and dual X-ray absorptiometry (DEXA) to evaluate body fat. CT determines intra-abdominal fat and subcutaneous fat [5]. Due to the high cost and technical problems, this method is not suitable for checking the amount of body fat in the general population [7]. DEXA can determine total body fat and regional fat; however, it is not cost-effective [4]. Currently, body mass index (BMI) is recommended by the World Health Organization (WHO) to check overweight and obesity in the general population. BMI does not give us information about central obesity or visceral fat. Therefore, methods such as measuring WC and waist-to-hip ratio (WHR) are used to measure central obesity. The size of the waist is related to factors such as the rate of breathing and the size of the stomach after eating and is very different in individuals. NC measurement does not have these mentioned disadvantages and can be used to classify people with normal weight and obese people. NC is a simple screening method that can be used as an indicator of upper body fat distribution to identify obesity [5]. In 2012, in a cross-sectional study, Tibana et al. investigated the relationship between NC and relative muscle strength and cardiovascular risk factors in 60 sedentary women [8]. The results showed that women with higher NC equal to 35 cm have more cardiovascular risk factors and lower relative muscle strength. NC can be used as an indicator of upper body fat measurement also due to its ease of use.

In addition, physical activity programs with an emphasis on muscle strength were suggested to prevent and treat increased NC and reduce the risk factors of cardiovascular diseases. The results of Tatar et al.'s study showed that NC has a positive and significant relationship with age, weight, WHR, BMI, hip circumference (HC), WC, insulin level, and insulin resistance index; a negative relationship with adiponectin and high-density lipoprotein cholesterol (HDL-C) in women; and a significant positive relationship with weight, BMI, HC, WC, and adiponectin in men. Similarly, this research showed that there is a positive and significant correlation of WC with weight, HP WHR, BMI, NC, insulin, and insulin resistance index, and a negative correlation with adiponectin and HDL-C only in women. In contrast, no significant relationship was observed between the WC with the parameters studied in men. Researchers concluded that NC can be a reliable indicator of obesity and insulin resistance in both genders [9]. By studying the relationship between NC and cardiovascular risk factors in 2019, Ben-Noun and Laor showed that NC in men has a significant direct relationship with LDL, TG, very low-density lipoprotein (VLDL), and ratio of total cholesterol (TC) to HDL, and in women, it has a direct and significant relationship with the ratio of TC to HDL, triglyceride, and VLDL. They concluded that neck size has a positive and significant correlation with cardiovascular risk factors in middle-aged men and women. Therefore, this new anthropometric index can be used as a simple and accurate tool to detect the risk of cardiovascular diseases in these groups of individuals [10]. This cross-sectional (descriptive-analytical) study was based on the data of the Bushehr Geriatric Health Cohort Study and a cohort study conducted in three phases to investigate the relationship between NC and CMRs in the elderly people.

## Materials and methods

In the first and second phases of the study, which was complementary, individuals older than 60 years in Bushehr residing in the study were enrolled. The form of physical and anthropometric examination such as height, weight, WC, NC, HC, WHR, BMI, body adiposity index (BAI), A body shape index (ABSI), abdominal volume index (AVI), and waist triglyceride index (WTI) were completed. In the first phase, the call method was door-to-door, and in the second phase, it was phone calls (according to the first phase information). In both phases, the participants entered the study with informed consent, and the exclusion criterion was not living in Bushehr port due to immigration or any other reason. The first phase was conducted from March 2012 to March 2014 with a number of 3,000 participants over 60 years old (91% coverage) in two phases (2.5 years in total), and the second phase from March 2015 to March 2017 with a number of 2,426 participants (with 92% coverage) was conducted again in two stages (2.5 years in total). Risk factors have been and will be evaluated every five years in three phases and for a total of 15 years with similar methods. In the first phase, the sampling process was stopped for two months, in the month of Ramadan and one month after, due to possible hemodynamic and biochemical changes that are the result of fasting, which made our measurement valid and reliable. An invitation letter was sent to all eligible elderly people to participate in the study. The criteria for entering the study include the age of 60 years and older, residing in Bushehr port for at least one year before entering the project, not having plans to leave Bushehr within two years after entering the project, having the sufficient physical and mental strength to participate in the evaluation program, and having full consent to participate in the study. Failure to reside in Bushehr, unwillingness to participate in the investigation, and death were considered exclusion criteria.

Every year, all participants were contacted and a checklist was completed to check the required data. All hospitals in Bushehr port were asked to report the cases that affect the information and also the results to the study authorities. The focal point of this study was responsible for the daily review of the Hospital Information Registration System (HIS) and ensuring the quality of the reports. If any results were reported, a general practitioner would review the medical records, and the detailed information would be entered in special forms. The database of the death registration system, available in the public health system, and the record system records information from hospitals, forensic departments, cemeteries, and critical incident offices, and duplicate files were deleted. Also, the ICD-10 coding system was used to classify the causes of death. If any death was reported, which was not confirmed by a valid death certificate, an autopsy was performed to determine the cause of death.

Ethics approval

The study protocol was approved by the ethics committee of the Endocrinology and Metabolism Research Institute, which is affiliated with Tehran University of Medical Science as well as the Research Ethics Committee of Bushehr University of Medical Sciences in 2021 (References number IR.BPUMS.REC.1400.061).

Society and research unit

The participants include all women and men over 60 years of age and living in Bushehr city, based on the information available in the health center of Bushehr city in 2011, when the first phase of the study was conducted and the population of Bushehr city over 60 years old was 10,000. Among them, 3,000 people were selected in the first phase of the study and 2,426 people were selected in the second phase of the study.

Sampling

Multi-stage stratified cluster random sampling based on the classified method of management and planning organization of Bushehr Province was conducted on 75 floors, and the sample size for the floors was determined according to the number of households residing on each floor. Each block consisted of several clusters of houses, which were separated from other houses by alleys or passages. Three blocks were eliminated since they were situated on military bases (the army air base and naval base) and the Halileh area, which was outside the municipal limits, making a total of 78 blocks. Hence, a total of 75 blocks were determined as sampling areas. 

Demographic Information Check Form

This form consists of two parts. First part is the demographic information, which includes name, nickname, last name, national number, age, gender, marital status, and contact information. The second part is the socio-economic information, which includes education level and marital status.

Physical and Clinical Examination Form

The physical examination form includes anthropometric measurements such as height and weight, WC, HC, WHR, BMI, BAI, ABSI, AVI, and WTI.

Measurement of Height and Weight

A comprehensive physical examination including vital signs and the measurement of weight, height, WC, and HC were performed at the beginning of work. The external ear hole should be along the lower edge of the eye socket. To measure the weight, the Seka digital device approved by the WHO was used.

Measuring WC

To measure WC, a person stands and looks straight ahead. The tape measure is closed at the narrowest point of the waist, in the navel area. While the person is breathing normally and has not held the breath in the chest, the corresponding number is recorded in centimeters.

Measurement of the HC

The person stands straight and looks in front. The weight should be evenly distributed on both legs. The tape measure is closed around the widest point of the buttocks, the most prominent point of the gluteus muscle. The meter should be completely horizontal both in front and back. The corresponding number is recorded in centimeters.

NC Measurement

The NC was measured in a horizontal line that passes right below Adam's apple in a state where the head is not bent forward, backward, or sideways.

Blood Pressure Measurement

Blood pressure was measured by an Omron arm digital sphygmomanometer (Langenfeld, Germany). After 15 minutes of rest, two blood pressures (two diastolic blood pressure [DBP] and two systolic blood pressure [SBP]) were taken from each participant at an interval of 10 minutes and then their average was determined as his blood pressure.

Measurement of Diabetes Status

To determine diabetic people, three self-reported methods, HBa1C, and FBG were used so that if one of these options is positive in a person, we identified him as a diabetic person. Participants adhered to the sampling site fasting (from 8 to 12 hours). A tube containing ethylenediaminetetraacetic acid (EDTA) was used to perform complete blood count (CBC) and HBA1c tests. It has been one of the primary trials of FBG participants.

Measuring the State of Metabolic Syndrome

To evaluate obesity, which is one of the indicators of metabolic syndrome, various methods were used, including BMI, NC, WC, and HC. WHR and AVI were also used.

In this study, BMI, WC, HC, and WHR were used for evaluation. Also, to check blood pressure, blood lipids, and glucose tolerance, the clinical method of sphygmomanometer and laboratory methods of TC, LDL cholesterol, HDL cholesterol, TG, FBG, and HbA1C were used.

In order to diagnose metabolic syndrome, the indicators of WC, blood pressure, TG, HDL cholesterol, and blood glucose were used. If three of the five indicators mentioned in Table 1 are higher than the normal cut-off range, the person is considered to have metabolic syndrome.

**Table 1:** How to diagnose metabolic syndrome HDL, high-density lipoprotein

Indicator	Natural cut-off
Waist	Men less than 102 cm
	Women less than 88 cm
Triglyceride	Less than 150 mm/gL
HDL cholesterol	Men more than 40 mm/gL
	Women more than 50 mm/gL
Blood pressure	Systolic blood pressure less than 130 mmHg
	Diastolic blood pressure less than 85 mmHg
Blood glucose	More than 100 mm/gL

Data analysis method

Quantitative variables will be described using their mean and standard deviation. Categorized data are presented as frequency and percentage. The strength of relationships between potential effects and risk factors was demonstrated by calculating ratios, risk and odds, and 95% confidence intervals. Correlation tests were used to check the relationships between variables. Since the dependent variables under investigation are two-mode, simple univariate regression and multivariate regression were used to obtain the raw odds ratio and adjusted odds ratio, and the STATA MP Version 15 software (StataCorp, College Station, TX) was used for data analysis.

## Results

The results of this study show that in the first phase of the study, the mean and standard deviation of age in men, women, and the entire study population were 68.07 ± 7.17, 67.65 ± 7.03, and 67.75 ± 7.10, respectively, and in the second phase of the study, they were 69.54 ± 6.44, 69.16 ± 6.35, and 69.34 ± 6.39, respectively. In general, the average age was higher in men than women, but this difference was not significant. Among the anthropometric indices, NC was measured only in the second phase of the study, and the results showed that in men, women, and all participants, the mean and standard deviation were 39.31 ± 2.89, 34.86 ± 2.84, and 37.00 ± 3.62, respectively.

The NC index was higher in men than in women, and this difference was significant (P-value < 0.001). Other anthropometric indices, including weight (in both phases), height (in both phases), and WC in the first phase, ABSI in the second phase, WHR in both phases, and DBS in the second phase, the mean and standard deviation were significantly higher in men (P-value < 0.001). Also, BMI and WC in the second phase, HC in both phases, AVI in the second phase, WTI in the second phase, and BAI were significantly higher in both phases in women (P-value < 0.001).

In most of the laboratory indicators including TG, TC, LDL, and HDL, the mean and standard deviation were significantly higher in women (P-value < 0.001). There was no significant difference in FBG between the male and female groups. Also, in the first phase of the study, 47.91%, 61.18%, and 45.96% of men, women, and all participants, respectively, were suffering from metabolic syndrome. In the second phase of the study, 44.33%, 57.67%, and 51.20% of men, women, and all participants, respectively, were suffering from metabolic syndrome, which was higher than in the first phase overall. The descriptive results can be seen in full in Table 2.

**Table 2:** Demographic characteristics of the participants in the Bushehr aging health study by gender (male and female) and overall NC, neck circumference; WC, waist circumference; HC, hip circumference; ABSI, A body shape index; WHR, waist-to-hip ratio; AVI, abdominal volume index; WTI, waist triglyceride index; BAI, body adiposity index; SBP, systolic blood pressure; DBP, diastolic blood pressure; FBG, fast blood glucose; TG, triglyceride; TC, total cholesterol; LDL-C, low-density lipoprotein cholesterol; HDL-C, high-density lipoprotein cholesterol; MetS, metabolic equivalents *Mann-Whitney U Test

Grouping	Variable	Phase	Men (N = 1,455)	Women (N = 1,545)	Total (N = 3,000)	P-value^*^
	Age (years)	Phase 1	68.07 ± 7.17	67.65 ± 7.03	67.75 ± 7.10	0.11
		Phase 2	69.54 ± 6.44	69.16 ± 6.35	69.34 ± 6.39	0.14
Anthropometric	Weight (kg)	Phase 1	71.77 ± 12.51	66.24 ± 13.19	38.93 ± 13.16	<0.001
		Phase 2	72.30 ± 12.39	66.60 ± 13.12	69.34 ± 13.09	<0.001
	Height (cm)	Phase 1	166.21 ± 6.51	153.05 ± 6.41	159.46 ± 9.22	<0.001
		Phase 2	165.87 ± 6.30	152.24 ± 6.12	158.79 ± 9.21	<0.001
	BMI (kg/m2)	Phase 1	25.94 ± 4.07	28.25 ± 5.34	27.12 ± 4.90	<0.001
		Phase 2	26.23 ± 4.01	28.70 ± 5.33	27.51 ± 4.90	<0.001
	NC (cm)	Phase 2	39.31 ± 2.89	34.86 ± 2.84	37.00 ± 3.62	<0.001
	WC (cm)	Phase 1	91.34 ± 8.88	89.70 ± 10.54	90.50 ± 9.80	<0.001
		Phase 2	97.08 ± 11.22	100.22 ± 12.52	98.71 ± 12.01	<0.001
	HC (cm)	Phase 1	98.80 ± 7.06	103.36 ± 10.67	101.14 ± 9.37	<0.001
		Phase 2	99.33 ± 7.67	105.56 ± 11.20	102.56 ± 10.16	<0.001
	ABSI	Phase 1	0.22 ± 8.39	0.01 ± 0.24	0.11 ± 5.85	0.32
		Phase 2	0.003 ± 0.0008	0.002 ± 0.0008	0.003 ± 0.0009	<0.001
	WHR	Phase 1	0.92 ± 0.05	0.87 ± 0.15	0.89 ± 0.11	<0.001
		Phase 2	0.97 ± 0.09	0.94 ± 0.07	0.96 ± 0.08	<0.001
	AVI	Phase 1	33.68 ± 6.48	33.91 ± 50.50	33.80 ± 36.44	0.86
		Phase 2	38.20 ± 8.61	40.80 ± 9.94	39.55 ± 9.41	<0.001
	WTI	Phase 1	147.01 ± 85.28	152.24 ± 86.33	149.70 ± 85.85	0.09
		Phase 2	144.47 ± 80.88	161.07 ± 86.52	153.08 ± 84.25	<0.001
	BAI	Phase 1	28.20 ± 3.86	36.73 ± 6.30	32.57 ± 6.76	<0.001
		Phase 2	28.57 ± 3.93	38.32 ± 6.55	33.63 ± 7.31	<0.001
	SBP (mmHg)	Phase 1	134.27 ± 18.44	135.43 ± 20.20	134.87 ± 19.37	0.10
		Phase 2	140.05 ± 19.44	139.28 ± 19.20	139.65 ± 19.32	0.32
	DBP (mmHg)	Phase 1	76.98 ± 12.18	76.80 ± 8.23	76.88 ± 10.33	0.62
		Phase 2	82.40 ± 8.74	80.76 ± 8.52	81.55 ± 8.67	<0.001
Laboratory	FBG (mg/dL)	Phase 1	109.24 ± 50.67	110.77 ± 46.05	110.03 ± 48.35	0.38
		Phase 2	104.57 ± 40.30	107.75 ± 44.55	106.22 ± 42.58	0.06
	TG (mg/dL)	Phase 1	140.45 ± 75.68	147.73 ± 74.76	144.20 ± 75.37	0.008
		Phase 2	130.26 ± 68.27	141.16 ± 72.01	135.92 ± 70.43	<0.001
	TC (mg/dL)	Phase 1	189.97 ± 43.70	207.29 ± 48.03	198.88 ± 46.78	<0.001
		Phase 2	173.31 ± 43.70	190.40 ± 45.50	182.18 ± 44.14	<0.001
	LDL-C (mg/dL)	Phase 1	117.64 ± 36.92	127.84 ± 41.57	122.98 ± 39.70	<0.001
		Phase 2	104.59 ± 34.79	113.91 ± 39.70	109.43 ± 37.70	<0.001
	HDL-C (mg/dL)	Phase 1	44.12 ± 14.23	49.17 ± 11.63	46.72 ± 13.20	<0.001
		Phase 2	43.08 ± 10.12	48.58 ± 11.52	45.94 ± 11.21	<0.001
	MetS	Phase 1	Yes: 606 (47.91)	Yes: 878 (61.18)	Yes: 1,484 (55.96)	-
			No: 659 (52.09)	No: 557 (38.82)	No: 1216 (45.04)	
		Phase 2	Yes: 645 (44.33)	Yes: 891 (57.67)	Yes: 1,536 (51.20)	-
			No: 810 (55.67)	No: 654 (42.33)	No: 1,464 (48.80)	

In overweight and non-overweight people, the mean and standard deviation of the NC index were 37.48 ± 3.43 and 36.66 ± 3.72, respectively, and this difference was significant. In people with and without obesity, the mean and standard deviation of the NC index were 38.36 ± 3.57 and 36.51 ± 3.51, respectively, and this difference was significant. In individuals with high and normal SBP, the mean and standard deviation of the NC index were 37.47 ± 3.60 and 36.60 ± 3.59, respectively, and this difference was significant (P-value < 0.001). In participants with high and normal DBP, the mean and standard deviation of the NC index were 37.84 ± 3.60 and 36.73 ± 3.59, respectively, and this difference was significant (P-value < 0.001). In people with high and normal FBG, the mean and standard deviation of the NC index were 37.84 ± 3.68 and 36.83 ± 3.59, respectively, and this difference was significant (P-value < 0.001). In individuals with high and normal blood TG, the mean and standard deviation of the NC index were 37.43 ± 3.48 and 36.80 ± 3.67, respectively, and this difference was significant (P-value < 0.001).

In participants with high and normal TC, the mean and standard deviation of the NC index were 36.47 ± 3.51 and 37.26 ± 3.65, respectively, which were significantly higher in the group with normal TG. In people with high and normal LDL cholesterol, the mean and standard deviation of the NC index were 36.73 ± 3.58 and 37.24 ± 3.64, respectively, which were significantly higher in the group with normal LDL cholesterol. In participants with high and normal HDL cholesterol, the mean and standard deviation of the NC index were 36.53 ± 3.56 and 37.87 ± 3.57, respectively, which was significantly higher in the group with normal HDL cholesterol (P-value < 0.001). In people with and without metabolic syndrome, the mean and standard deviation of the NC index were 37.39 ± 3.58 and 36.33 ± 3.60, respectively, which were significantly higher in people with metabolic syndrome (P-value < 0.001). Information about other body composition indicators can be seen in detail in Table 3.

**Table 3:** Mean and standard deviation of anthropometric profiles by metabolic syndrome factors in the Bushehr aging health study participants NC, neck circumference; WC, waist circumference; HC, hip circumference; ABSI, A body shape index; WHR, waist-to-hip ratio; AVI, abdominal volume index; WTI, waist triglyceride index; BAI, body adiposity index; SBP, systolic blood pressure; DBP, diastolic blood pressure; FBG, fast blood glucose; TG, triglyceride; TC, total cholesterol; LDL-C, low-density lipoprotein cholesterol; HDL-C, high-density lipoprotein cholesterol; MetS, metabolic equivalents *Mann-Whitney U test

Variable	Phase		NC	WC	HC	ABSI	WHR	AVI	WTI	BAI
Underweight	Phase 1	Yes	-	70.41 ± 6.14	85.10 ± 4.99	0.005 ± 0.0009	0.82 ± 0.05	19.97 ± 3.52	72.13 ± 23.76	23.45 ± 3.44
		No	-	91.98 ± 8.14	101.52 ± 9.12	0.003 ± 0.0008	0.89 ± 0.12	34.13 ± 36.85	151.61 ± 86.02	33.78 ± 6.68
		^P-value*^	-	<0.001	<0.001	<0.001	<0.001	<0.001	<0.001	<0.001
	Phase 2	Yes	32.76 ± 2.623.59	71.51 ± 6.83	84.81 ± 3.70	0.005 ± 0.001	0.84 ± 0.06	20.62 ± 3.94	25.17 ± 70.72	24.28 ± 4.12
		No	37.09 ± 3.59	89.64 ± 11.60	102.92 ± 9.92	0.003 ± 0.0008	0.96 ± 0.08	39.93 ± 9.09	154.75 ± 84.19	33.82 ± 7.24
		P-value	<0.001	<0.001	<0.001	<0.001	<0.001	<0.001	<0.001	<0.001
Overweight	Phase 1	Yes	-	91.77 ± 6.07	101.37 ± 4.69	0.002 ± 0.0003	0.90 ± 0.06	33.82 ± 4.44	159.26 ± 87.00	32.39 ± 4.61
		No	-	89.64 ± 11.60	100.99 ± 11.52	0.19 ± 7.60	0.89 ± 0.14	33.78 ± 47.15	143.17 ± 84.46	32.69 ± 7.91
		P-value	-	<0.001	0.22	0.38	<0.001	0.97	<0.001	0.19
	Phase 2	Yes	37.48 ± 3.43	99.32 ± 6.82	102.08 ± 5.21	0.003± 0.0003	0.97 ± 0.07	39.64 ± 5.23	158.50 ± 83.31	32.81 ± 4.91
		No	36.66 ± 3.72	98.27 ± 14.69	102.93 ± 12.60	0.003 ± 0.0001	0.95 ± 0.09	39.49 ± 11.57	149.09 ± 84.73	34.23 ± 8.61
		P-value	<0.001	0.01	0.02	<0.001	<0.001	0.69	0.006	<0.001
Obesity	Phase 1	Yes	-	100.38 ± 7.56	112.19 ± 8.21	0.002 ± 0.0005	0.90 ± 0.21	43.21 ± 72.43	179.39 ± 96.26	39.60 ± 6.88
		No	-	94.33 ± 9.58	97.59 ± 6.50	0.003 ± 0.0008	0.89 ± 0.06	30.77 ± 5.62	140.28 ± 80.04	30.30 ± 4.91
		P-value	-	<0.001	<0.001	<0.001	0.07	<0.001	<0.001	<0.001
	Phase 2	Yes	38.36 ± 3.57	110.83 ± 9.47	113.87 ± 9.41	0.002 ± 0.0004	0.97 ± 0.11	49.49 ± 8.09	185.97 ± 89.70	40.84 ± 7.51
		No	36.51 ± 3.51	94.33 ± 9.58	98.47 ± 6.74	0.003 ± 0.0007	0.95 ± 0.07	35.95 ± 6.95	141.18 ± 78.90	31.02 ± 5.18
		P-value	<0.001	<0.001	<0.001	<0.001	<0.001	<0.001	<0.001	<0.001
SBP	Phase 1	High	-	91.96 ± 9.66	102.15 ± 9.71	0.009 ± 0.22	0.90 ± 0.06	34.20 ± 7.15	159.36 ± 91.14	33.41 ± 7.10
		Normal	-	89.61 ± 9.78	100.52 ± 9.10	0.18 ± 7.43	0.89 ± 0.14	33.56 ± 45.92	143.79 ± 81.91	32.06 ± 6.50
		P-value	-	<0.001	<0.001	0.44	0.17	0.64	<0.001	<0.001
	Phase 2	High	37.47 ± 3.60	100.64 ± 10.38	103.34 ± 10.38	0.003 ± 0.0009	0.97 ± 0.07	41.06 ± 9.47	162.58 ± 86.42	34.04 ± 7.35
		Normal	36.60 ± 3.59	97.08 ± 12.00	101.90 ± 9.92	0.003 ± 0.0008	0.95 ± 0.02	38.27 ± 9.17	145.04 ± 81.53	33.28 ± 7.26
		P-value	<0.001	<0.001	<0.001	<0.001	<0.001	<0.001	<0.001	<0.001
DBP	Phase 1	High	-	93.47 ± 10.30	103.73 ± 11.22	0.002 ± 0.0008	0.90 ± 0.06	35.36 ± 7.84	166.00 ± 96.25	33.97 ± 7.60
		Normal	-	90.31 ± 9.74	100.97 ± 9.22	0.12 ± 6.04	0.89 ± 0.12	33.70 ± 37.53	148.66 ± 85.06	33.48 ± 6.70
		P-value	-	<0.001	<0.001	0.79	0.54	0.55	0.009	0.004
	Phase 2	High	37.84 ± 3.60	100.88 ± 12.56	103.84 ± 10.69	0.003 ± 0.0008	0.97 ± 0.08	41.33 ± 10.03	162.36 ± 83.86	33.60 ± 7.62
		Normal	36.73 ± 3.59	98.03 ± 11.75	102.16 ± 9.95	0.003 ± 0.0009	0.96 ± 0.8	38.99 ± 9.14	150.16 ± 84.18	33.64 ± 7.21
		P-value	<0.001	<0.001	<0.001	<0.001	0.002	<0.001	0.002	0.89
FBG	Phase 1	High	-	93.15 ± 9.14	101.80 ± 9.85	0.01 ± 0.31	0.92 ± 0.23	38.38 ± 80.86	193.68 ± 113.24	32.80 ± 7.36
		Normal	-	89.86 ± 9.85	100.98 ± 9.24	0.13 ± 6.53	0.89 ± 0.06	32.68 ± 7.12	138.95 ± 73.68	32.51 ± 6.61
		P-value	-	<0.001	0.59	0.64	<0.001	<0.001	<0.001	0.35
	Phase 2	High	37.84 ± 3.68	101.53 ± 11.31	103.50 ± 10.42	0.003 ± 0.0009	0.98 ± 0.06	41.74 ± 9.34	197.55 ± 106.94	34.01 ± 7.43
		Normal	36.83 ± 3.59	98.13 ± 12.07	102.37 ± 10.09	0.003 ± 0.0007	0.95 ± 0.08	39.10 ± 9.37	143.87 ± 75.55	33.55 ± 7.28
		P-value	<0.001	<0.001	0.03	0.006	<0.001	<0.001	<0.001	0.24
TG	Phase 1	High	-	92.87 ± 8.99	102.63 ± 9.06	0.01 ± 0.23	0.91 ± 0.17	36.65 ± 60.12	230.34 ± 93.96	33.39 ± 6.86
		Normal	-	89.19 ± 9.98	100.31 ± 9.44	0.17 ± 7.30	0.89 ± 0.06	32.21 ± 7.17	104.91 ± 30.71	32.12 ± 6.67
		P-value	-	<0.001	<0.001	0.46	<0.001	<0.001	<0.001	<0.001
	Phase 2	High	37.43 ± 3.48	101.36 ± 11.05	104.31 ± 10.00	0.003 ± 0.0007	0.97 ± 0.06	41.58 ± 8.93	245.30 ± 85.70	34.64 ± 7.50
		Normal	36.80 ± 3.67	97.49 ± 12.24	101.76 ± 10.13	0.003 ± 0.0009	0.95 ± 0.09	38.61 ± 9.49	110.29 ± 34.73	33.16 ± 7.18
		P-value	<0.001	<0.001	<0.001	<0.001	<0.001	<0.001	<0.001	<0.001
TC	Phase 1	High	-	90.52 ± 9.84	101.59 ± 9.68	0.23 ± 8.45	0.89 ± 7.13	33.16 ± 7.13	168.44 ± 88.89	33.30 ± 6.90
		Normal	-	90.48 ± 9.76	100.73 ± 9.06	0.006 ± 0.14	0.90 ± 0.15	34.39 ± 50.09	132.34 ± 79.09	31.90 ± 6.56
		P-value	-	0.92	0.01	0.29	0.02	0.36	<0.001	<0.001
	Phase 2	High	36.47 ± 3.51	98.98 ± 11.86	103.42 ± 10.51	0.003 ± 0.0008	0.95 ± 0.07	39.74 ± 9.28	181.57 ± 96.73	34.93 ± 7.30
		Normal	37.26 ± 3.65	38.59 ± 12.09	102.15 ± 9.96	0.003 ± 0.0009	0.96 ± 0.09	39.46 ± 9.48	139.20 ± 73.51	33.00 ± 7.23
		P-value	<0.001	0.45	0.003	<0.001	0.02	0.48	<0.001	<0.001
LDL-C	Phase 1	High	-	90.42 ± 9.73	101.22 ± 9.38	0.18 ± 7.53	0.89 ± 0.06	33.08 ± 7.06	153.94 ± 76.14	32.84 ± 6.70
		Normal	-	90.63 ± 9.90	101.01 ± 9.36	0.008 ± 0.16	0.90 ± 0.17	34.90 ± 57.33	143.19 ± 98.59	32.16 ± 6.84
		P-value	-	0.57	0.55	0.42	0.06	0.18	<0.001	0.08
	Phase 2	High	36.73 ± 3.58	98.63 ± 11.85	102.71 ± 10.11	0.003 ± 0.0008	0.96 ± 0.07	39.47 ± 9.28	160.57 ± 81.60	34.02 ± 7.20
		Normal	37.24 ± 3.64	98.80 ± 12.16	102.44 ± 10.21	0.003 ± 0.0009	0.96 ± 0.09	39.63 ± 9.54	146.48 ± 86.02	33.28 ± 7.40
		P-value	<0.001	0.72	0.52	0.03	0.15	0.66	<0.001	0.01
HDL-C	Phase 1	High	-	89.55 ± 10.11	101.12 ± 9.84	0.17 ± 7.17	0.88 ± 0.13	33.47 ± 44.35	131.78 ± 71.91	33.02 ± 6.97
		Normal	-	92.42 ± 8.85	101.18 ± 8.35	0.003 ± 0.0008	0.91 ± 0.06	34.47 ± 6.60	185.79 ± 99.28	31.66 ± 6.22
		P-value	-	<0.001	0.88	0.46	<0.001	0.48	<0.001	<0.001
	Phase 2	High	36.53 ± 3.56	98.31 ± 12.33	102.86 ± 10.73	0.003 ± 0.0009	0.95 ± 0.09	39.26 ± 9.62	134.95 ± 66.63	34.34 ± 7.56
		Normal	37.87 ± 3.57	99.46 ± 11.38	102.03 ± 9.00	0.003 ± 0.0009	0.97 ± 0.07	40.08 ± 9.00	186.27 ± 101.33	32.33 ± 6.63
		P-value	<0.001	0.02	0.055	0.03	<0.001	0.04	<0.001	<0.001
MetS	Phase 1	High	-	93.87 ± 8.66	103.56 ± 9.13	0.008 ± 0.19	0.91 ± 0.15	36.86 ± 51.13	192.42 ± 96.93	34.07 ± 6.92
		Normal	-	88.79 ± 9.57	99.88 ± 9.16	0.008 ± 0.16	0.89 ± 0.06	31.89 ± 6.79	111.35 ± 42.89	31.66 ± 6.42
		P-value	-	<0.001	<0.001	0.99	<0.001	<0.001	<0.001	<0.001
	Phase 2	High	37.39 ± 3.58	101.69 ± 10.47	104.39 ± 9.82	0.003 ± 0.0007	0.97 ± 0.07	41.80 ± 8.62	182.77 ± 89.72	34.84 ± 7.33
		Normal	36.33 ± 3.60	93.56 ± 12.74	99.42 ± 9.96	0.003 ± 0.001	0.94 ± 0.10	35.66 ± 9.45	101.86 ± 36.22	31.54 ± 6.79
		P-value	<0.001	<0.001	<0.001	<0.001	<0.001	<0.001	<0.001	<0.001

Table 4 and Table 5 show the linear correlation of anthropometric indices with metabolic syndrome risk factors in the first and second phases, respectively. In the second phase of the study, the NC index in the total population was significantly associated with all metabolic syndrome risk factors, including BMI (R=0.356), SBP (R=0.160), DBP (R=0.186), FBG (R= 0.078), and TG (R=0.085), and it also has a direct and significant relationship with risk factors of metabolic syndrome including TC, low-density lipoprotein cholesterol (LDL-C), and HDL-C; as the size of the NC increases, these indices decrease. However, all relationships showed a significant but weak relationship. Also, in the male population, the NC index is definitely and significantly related to BMI (R = 0.757), SBP (R= 0.205), DBP (R= 0.197), and FBG (R= 0.136) index, and HDL-C is inversely significant. In the male population, there was no correlation between the NC index and the TC and LDL-C indices. Also, in the female population, the NC index is definitely and significantly related to BMI (R=0.614), SBP (R=0.171), DBP (R=0.130), and FBG (R=0.122), and is inversely and significantly related to HDL-C and LDL-C index. In the female population, there was no correlation between the NC index and the TC index.

**Table 4:** Correlation of anthropometric indices with metabolic syndrome factors in the participants in the Bushehr aging health study in the first phase WC, waist circumference; HC, hip circumference; ABSI, A body shape index; WHR, waist-to-hip ratio; AVI, abdominal volume index; WTI, waist triglyceride index; BMI, body mass index; SBP, systolic blood pressure; DBP, diastolic blood pressure; FBG, fasting blood glucose; TG, triglyceride; TC, total cholesterol; LDL-C, low-density lipoprotein cholesterol; HDL-C, high-density lipoprotein cholesterol Note: Spearman correlation for all cells in the table

Risk factors of metabolic syndrome		BMI	SBP	DBP	FBG	TG	TC	LDL-C	HDL-C
WC	Total	0.792	0.175	0.149	0.100	0.195	0.009	-0.013	-0.147
	Men	0.858	0.199	0.115	0.102	0.248	0.031	0.005	-0.170
	Female	0.827	0.162	0.205	0.103	0.162	0.021	-0.008	-0.102
HC	Total	0.864	0.123	0.126	0.006	0.118	0.055	0.020	0.003
	Men	0.817	0.143	0.104	-0.012	0.137	0.012	0.005	-0.099
	Female	0.877	0.109	0.183	0.014	0.096	0.011	-0.021	-0.007
ABSI	Total	-0.856	-0.029	-0.014	-0.013	-0.016	0.007	0.014	0.002
	Men	-0.911	-0.042	-0.017	-0.013	-0.021	0.016	0.026	0.006
	Female	-0.838	-0.015	-0.015	0.003	-0.021	0.010	0.006	0.045
WHR	Total	0.077	0.050	0.030	0.089	0.081	-0.042	-0.040	-0.121
	Men	0.415	0.153	0.060	0.178	0.234	0.037	0.004	-0.156
	Female	0.065	0.031	0.023	0.078	0.056	-0.015	-0.020	-0.070
AVI	Total	0.180	0.024	0.025	0.028	0.040	-0.014	-0.023	-0.027
	Men	0.860	0.197	0.114	0.099	0.243	0.028	0.001	-0.166
	Female	0.152	0.010	0.025	0.028	0.027	-0.023	-0.032	-0.027
WTI	Total	0.292	0.128	0.0103	0.222	0.952	0.291	0.110	-0.121
	Men	0.325	0.154	0.099	0.146	0.986	0.312	0.125	-0.156
	Female	0.274	0.104	0.114	0.300	0.921	0.272	0.092	-0.070

**Table 5:** Correlation of anthropometric indices with metabolic syndrome factors in the participants in the Bushehr aging health study in the second phase NC, neck circumference; WC, waist circumference; HC, hip circumference; ABSI, A body shape index; WHR, waist-to-hip ratio; AVI, abdominal volume index; WTI, waist triglyceride index; BMI, body mass index; SBP, systolic blood pressure; DBP, diastolic blood pressure; FBG, fast blood glucose; TG, triglyceride; TC, total cholesterol; LDL-C, low-density lipoprotein cholesterol; HDL-C, high-density lipoprotein cholesterol Note: Spearman correlation for all cells in the table

Risk factors of metabolic syndrome		BMI	SBP	DBP	FBG	TG	TC	LDL-C	HDL-C
NC	Total	0.365	0.160	0.186	0.078	0.085	-0.120	-0.093	-0.255
	Men	0.757	0.205	0.197	0.136	0.180	0.006	-0.018	-0.147
	Female	0.614	0.171	0.130	0.122	0.156	-0.010	-0.026	-0.128
WC	Total	0.824	0.193	0.174	0.100	0.168	0.019	-0.011	-0.091
	Men	0.866	0.204	0.205	0.129	0.195	0.021	-0.008	-0.148
	Female	0.802	0.193	0.176	0.072	0.133	-0.026	-0.042	-0.114
HC	Total	0.863	0.097	0.111	0.040	0.118	0.068	0.025	0.047
	Men	0.758	0.137	0.170	0.035	0.133	0.015	-0.010	-0.084
	Female	0.899	0.093	0.138	0.028	0.081	0.006	-0.015	0.002
ABSI	Total	-0.891	-0.133	-0.139	-0.052	-0.150	-0.113	-0.062	-0.047
	Men	-0.0891	-0.211	-0.232	-0.088	-0.189	-0.044	-0.019	0.141
	Female	-0.910	-0.094	-0.149	0.007	-0.078	-0.041	-0.014	-0.025
WHR	Total	0.207	0.159	0.128	0.094	0.098	-0.046	-0.041	-0.174
	Men	0.459	0.134	0.127	0.119	0.115	0.011	-0.002	-0.099
	Female	0.091	0.191	0.101	0.085	0.113	-0.049	-0.045	-0.194
AVI	Total	0.840	0.193	0.177	0.099	0.166	0.019	-0.012	-0.084
	Men	0.880	0.199	0.206	0.128	0.188	0.018	-0.010	-0.140
	Female	0.819	0.197	0.184	0.071	0.133	-0.026	-0.043	-0.112
WTI	Total	0.320	0.130	0.094	0.248	0.972	0.328	0.128	-0.360
	Men	0.350	0.113	0.135	0.218	0.979	0.340	0.133	-0.394
	Female	0.281	0.151	0.077	0.268	0.966	0.299	0.106	-0.402

The results of this study show that every 1-unit increase in the NC index increases the chance of obesity based on the BMI index by 1.21% in the crude analysis mode and by 1.73% in the adjusted analysis mode. Additionally, the risk of developing hypertension based on SBP is 1.06% in the crude analysis and 1.10% in the adjusted form, and based on DBP, it is 1.08% in the raw form and 1.06% in the adjusted mode, with each unit increase in the NC index. The NC index significantly increases the chance of developing diabetes; thus, with an increase of one unit in the NC index, the chance of developing type 2 diabetes increases by 1.04% in the raw state and 1.10% in the adjusted state. The NC index increases the chance of hypertriglyceridemia, but in the case of hypercholesterolemia, the results are the opposite and it decreases this chance. The results show that with each unit increase in NC, the chance of hypertriglyceridemia increases by 1.04% in the raw state and by 1.10% in the adjusted state. Also, with each unit increase in the size of the NC, the chance of having hypercholesterolemia decreases in the crude analysis mode; however, in the adjusted mode, this relationship is not significant. Regarding the LDL-C index, with each unit increase in NC, the chance of having high LDL-C decreases, but this relationship is not significant in the adjusted state. Also, with each unit increase in NC, the chance of having normal HDL-C decreases by 10% in the raw state and 7% in the adjusted state. In the end, it can be concluded that each unit increase in the NC index increases the chance of developing metabolic syndrome by 1.08% in the raw state and by 1.28% in the adjusted state. Table 6 shows the correlation between anthropometric indices and metabolic syndrome risk factors in univariate and multivariate logistic regression models in the participants in the Bushehr aging health study.

**Table 6:** Odds ratios of anthropometric indices with metabolic syndrome factors in univariate and multivariate logistic regression models in the participants in the Bushehr aging health study NC, neck circumference; WC, waist circumference; HC, hip circumference; ABSI, A body shape index; WHR, waist-to-hip ratio; AVI, abdominal volume index; WTI, waist triglyceride index; BAI, body adiposity index; BMI, body mass index; SBP, systolic blood pressure; DBP, diastolic blood pressure; FBG, fast blood glucose; TG, triglyceride; TC, total cholesterol; LDL-C, low-density lipoprotein cholesterol; HDL-C, high-density lipoprotein cholesterol; MetS, metabolic equivalents *Logistic regression

Variable	Phase	NC		WC		HC		ABSI		WHR		AVI		WTI		BAI	
		Raw OR	Justified OR	Raw OR	Justified OR	Raw OR	Justified OR	Raw OR	Justified OR	Raw OR	Justified OR	Raw OR	Justified OR	Raw OR	Justified OR	Raw OR	Justified OR
BMI	Phase 1	-	-	1.18 (1.17-1.20)	1.24 (1.21-1.26)	1.29 (1.26-1.31)	1.31 (1.28-1.35)	1.36 (0.55-3.39)	1.35 (0.44-4.08)	2.10 (1.78-2.47)	2.46 (2.03-2.99)	1.29 (1.26-1.31)	1.38 (1.34-1.42)	1.00	1.00	1.25 (1.23-1.28)	1.49 (1.44-1.55)
	Phase 2	1.21 (1.18-1.25)	1.73 (1.64-1.84)	1.16 (1.15-1.18)	1.17 (1.15-1.18)	1.28 (1.25-1.30)	1.28 (1.25-1.31)	-	-	2.93 (2.22-3.87)	2.45 (2.03-2.99)	1.24 (1.22-1.27)	1.25 (1.22-1.27)	1.00	1.00	1.21 (1.19-1.34)	1.45 (1.39-1.50)
SBP	Phase 1	-	-	1.02 (1.01-1.03)	1.02 (1.01-1.03)	1.01 (1.01-1.02)	1.02 (1.01-1.03)	0.98 (0.92-1.05)	0.98 (0.91-1.06)	1.46 (1.24-1.72)	1.45 (1.21-1.75)	1.00	1.00	1.00	1.00	1.02 (1.01-1.04)	1.03 (1.02-1.05)
	Phase 2	1.06 (1.04-1.09)	1.10 (1.07-1.14)	1.02 (1.01-1.03)	1.02 (1.01-1.03)	1.01 (1.01-1.02)	1.01 (1.01-1.02)	-	-	1.76 (1.32-2.36)	1.75 (1.28-2.39)	1.03 (1.02-1.04)	1.03 (1.02-1.04)	1.00	1.00	1.01 (1.00-1.02)	1.02 (1.00-1.04)
DBP	Phase 1	-	-	1.03 (1.02-1.04)	1.02 (1.01-1.04)	1.02 (1.01-1.04)	1.03 (1.01-1.05)	-	-	1.14 (0.81-1.60)	0.98 (0.67-1.42)	1.00	1.00	1.00	1.00	1.03 (1.00-1.05)	1.07 (1.03-1.10)
	Phase 2	1.08 (1.04-1.11)	1.06 (1.02-1.10)	1.02 (1.01-1.02)	1.01 (1.01-1.02)	1.01 (1.00-1.02)	1.01 (1.00-1.03)	-	-	1.43 (1.01-2.03)	1.43 (0.98-2.08)	1.02 (1.01-1.03)	1.03 (1.01-1.03)	1.00	1.00	0.99 (0.98-1.01)	1.02 (1.00-1.04)
FBG	Phase 1	-	-	1.03 (1.02-1.04)	1.03 (1.02-1.04)	1.00	1.00	0.99 (0.94-1.04)	0.99 (0.94-1.04)	2.22 (1.78-2.77)	2.31 (1.81-2.95)	1.04 (1.03-1.05)	1.03 (1.02-1.05)	1.00	1.00	1.00	0.99 (0.97-1.01)
	Phase 2	1.04 (1.02-1.07)	1.10 (1.06-1.14)	1.02 (1.02-1.03)	1.02 (1.01-1.03)	1.01 (1.00-1.02)	1.00 (0.99-1.01)	-	-	2.57 (1.57-4.21)	2.49 (1.47-4.22)	1.02 (1.01-1.04)	1.02 (1.01-1.03)	1.00	1.00	1.00 (0.99-1.02)	1.00 (0.99-1.02)
TG	Phase 1	-	-	1.04 (1.03-1.04)	1.03 (1.02-1.04)	1.02 (1.01-1.03)	1.01 (1.00-1.02)	0.98 (0.92-1.05)	0.98 (0.92-1.05)	1.75 (1.48-2.07)	1.85 (1.53-3.28)	1.05 (1.04-1.06)	1.04 (1.03-1.05)	1.11 (1.10-1.12)	1.11 (1.10-1.12)	1.02 (1.01-1.03)	1.02 (1.00-1.03)
	Phase 2	1.04 (1.02-1.07)	1.10 (1.06-1.14)	1.02 (1.02-1.03)	1.02 (1.01-1.03)	1.02 (1.01-1.03)	1.01 (1.00-1.02)	-	-	2.47 (1.72-3.55)	2.24 (1.53-3.28)	1.03 (1.02-1.04)	1.02 (1.01-1.03)	1.09 (1.08-1.10)	1.09 (1.08-1.10)	1.02 (1.01-1.03)	1.01 (1.00-1.03)
TC	Phase 1	-	-	1.00	1.00	1.00	1.00	1.04 (0.69-1.59)	1.02 (0.80-1.31)	0.91 (0.78-1.07)	0.94 (0.79-1.12)	0.99 (0.99-1.00)	0.99 (0.99-1.00)	1.00	1.00	1.03 (1.02-1.04)	1.00 (0.98-1.01)
	Phase 2	0.94 (0.91-0.96)	0.99 (0.96-1.02)	1.00	0.99 (0.99-1.00)	1.01 (1.00-1.02)	1.00	-	-	1.20 (0.89-1.63)	1.13 (0.82-1.57)	1.00	0.99 (0.98-1.00)	1.00	1.00	1.03 (1.02-1.04)	1.00
LDL-C	Phase 1	-	-	0.99 (0.99-1.00)	0.99 (0.99-1.00)	1.00	0.99 (0.98-1.00)	1.01 (0.90-1.13)	1.01 (0.91-1.13)	0.93 (0.79-1.12)	0.94 (0.79-1.12)	0.99 (0.98-1.00)	0.99 (0.98-1.00)	1.00	1.00	1.01 (1.00-1.02)	0.99 (0.98-1.01)
	Phase 2	0.96 (0.94-0.98)	0.99 (0.96-1.02)	0.99 (0.99-1.00)	0.99 (0.99-1.00)	1.00	1.00	-	-	1.04 (0.79-1.37)	1.04 (0.77-1.40)	0.99 (0.98-1.00)	0.99 (0.98-1.00)	1.00	1.00	1.01 (1.00-1.02)	1.00
HDL-C	Phase 1	-	-	0.97 (0.96-0.97)	0.97 (0.96-0.98)	0.99 (0.99-1.00)	0.99 (0.99-1.00)	-	-	0.54 (0.45-0.64)	0.56 (0.46-0.67)	0.99 (0.99-1.00)	0.99 (0.99-1.00)	0.99 (0.99-0.99)	0.99 (0.98-0.99)	1.03 (1.01-1.04)	0.99 (0.98-1.01)
	Phase 2	0.90 (0.88-0.92)	0.93 (0.90-0.96)	0.99 (0.98-0.99)	0.98 (0.97-0.99)	1.00	0.99 (0.98-1.00)	-	-	0.55 (0.40-0.76)	0.53 (0.37-0.75)	0.99 (0.98-0.99)	0.98 (0.97-0.99)	0.99 (0.99-0.99)	0.99 (0.98-0.99)	1.04 (1.02-1.05)	1.00
MetS	Phase 1	-	-	1.06 (1.05-1.07)	1.06 (1.05-1.07)	1.04 (1.03-1.05)	1.03 (1.02-1.04)	0.99 (0.65-1.51)	0.96 (0.63-1.47)	2.08 (1.75-2.46)	2.22 (1.82-2.71)	1.08 (1.07-1.10)	1.08 (1.06-1.10)	1.02 (1.02-1.02)	1.02 (1.02-1.02)	1.05 (1.04-1.06)	1.04 (1.02-1.06)
	Phase 2	1.08 (1.06-1.11)	1.28 (1.23-1.33)	1.06 (1.05-1.07)	1.06 (1.05-1.07)	1.05 (1.04-1.06)	1.04 (1.03-1.05)	-	-	3.60 (2.67-4.85)	4.58 (3.31-6.33)	1.08 (1.07-1.09)	1.07 (1.06-1.08)	1.02 (1.02-1.02)	1.02 (1.02-1.02)	1.07 (1.05-1.08)	1.06 (1.04-1.08)

## Discussion

In the first and second phases of the study, 47.96% and 51.20% of all participants in the study had metabolic syndrome, respectively, and the number of women with metabolic syndrome was more than men. In other studies, metabolic syndrome was reported to be 57.71% in the Iranian elderly in the north of the country [11], 9.72% in the Mexican elderly [12], 58.7% in the Spanish elderly and 58.7% in the Colombian elderly [13], and 44.45% in the Japanese elderly [14]. The difference in the prevalence of metabolic syndrome can be related to various factors such as age and gender distribution, different lifestyles in different geographies, and differences in diet and physical activity habits.

The results of this study showed that the average NC in men was significantly higher than that of women. Also, in most of the laboratory indicators, including blood TG, TC, LDL, and HDL, mean and standard deviation were significantly higher in women. There was no significant difference in FBG between the male and female groups. Therefore, it seems that the significant differences between men and women are due to differences in their physiology. A study conducted in Poland showed that women with abnormal cholesterol concentration had a similar anthropometric score, but men with high HDL concentration had a high anthropometric score [15].

 Moreover, in the second phase of the study, there were significant differences in the NC index between people who were and were not underweight, who were overweight, and had obesity, high SBP, high DBP, high FBG, high TG, high TC, high LDL cholesterol, high HDL, and metabolic syndrome. Also, the mean and standard deviation had a significant difference, and it was higher in the affected people.

In the second phase of the study, the NC index in the total population was significantly associated with all metabolic syndrome risk factors. Moreover, metabolic syndrome risk factors including TC, LDL-C, and HDL-C have an inverse and significant relationship with NC, and these indices decrease as the size of the NC increases.

Also, in the male population, the NC index is definitely and significantly related to BMI, SBP, DBP, and FBG indices, and is inversely related to the HDL-C index. In the male population, there was no correlation between the NC index and TC and LDL-C indices. Also, in the female population, the NC index is positively and significantly related to BMI, SBP, DBP, and FBG indices, and is inversely and significantly related to HDL-C and LDL-C indices. In the female population, there was no correlation between the NC index and the TC index.

NC increases the chance of obesity by 1.21% in the crude analysis and 1.73% in the adjusted analysis with every 1-unit increase. Additionally, the risk of developing hypertension based on SBP is 1.06% in the crude analysis and 1.10% in the adjusted form, and based on DBP, it is 1.08% in the raw form and 1.06% in the adjusted mode, with each unit increase in the NC index. The NC index significantly increases the chance of developing diabetes, so with an increase of one unit in the NC index, the chance of developing type 2 diabetes is 1.04% in the raw state and 1.10% in the adjusted state. These findings were consistent with previous findings [16-18].

The NC increases the chance of hypertriglyceridemia, which is consistent with previous studies [19]. However, in the case of hypercholesterolemia, this result was the opposite, and NC decreased the chance of hypertriglyceridemia. The results show that with each unit increase in NC, the chance of hypertriglyceridemia increases by 1.04% in the crude analysis and by 1.10% in the adjusted model. Also, the risk of hypercholesterolemia decreases by 6% with each unit rise in NC in the crude analysis mode, but this connection is not significant in the adjusted mode. As for the LDL-C index, with each unit increase in NC, the chance of having high LDL-C decreases by 4%, but this relationship becomes insignificant in the adjusted state.

Moreover, a unit increase in NC reduces the chance of having normal HDL-C by 10% in the crude analysis and 7% in the adjusted analysis. In the end, it can be concluded that each unit increase in the NC increases the chance of developing metabolic syndrome by 1.08% in the crude analysis and by 1.28% in the adjusted analysis. In general, it seems that the NC index has behaved similarly to BMI, blood pressure, and blood glucose indices in various studies and has increased the chance of infection, but the results are slightly different in the case of blood fat indices. The reason for this difference can be related to the studied population, the age of the people, the studied disease, and the geography of the studied place, which is different in different studies. In general, studies state that NC can be a simple and affordable indicator to diagnose metabolic syndrome in the elderly and even children.

As the limitations of this study, the effects of medications being taken by the participants and their dietary pattern on the metabolic syndrome were not considered in this study, and both of these variables can be a confounding factor in the relationship between metabolic syndrome and anthropometric indicators.

## Conclusions

The present study shows that the NC index can be a good predictor for the diagnosis of metabolic syndrome and visceral adipose tissue in the elderly. Factors such as gender, age, smoking habits, and reduced physical activity are significant risk factors in the development of metabolic syndrome in the elderly. Early diagnosis of metabolic syndrome reduces its complications and mortality and also reduces the risk of related diseases, including type 2 diabetes and cardiovascular diseases. Each unit increase in the NC index increases the chance of developing metabolic syndrome by 1.08% in the raw state and by 1.28% in the adjusted state.
